# Relaxin reduces xenograft tumour growth of human MDA-MB-231 breast cancer cells

**DOI:** 10.1186/bcr2136

**Published:** 2008-08-21

**Authors:** Yvonne Radestock, Cuong Hoang-Vu, Sabine Hombach-Klonisch

**Affiliations:** 1 Clinics of General, Visceral and Vascular Surgery, Magdeburger Str. 18, Martin-Luther-University Halle Wittenberg, 06097 Halle, Germany; 2 Departments of Human Anatomy and Cell Science, 130 Basic Medical Sciences, Winnipeg, MB, R3E 0W3, Canada; 3 Obstetrics and Gynecology, University of Manitoba, Faculty of Medicine, 130 Basic Medical Sciences, Winnipeg, MB, R3E 0W3, Canada

## Abstract

**Introduction:**

Relaxin levels are increased in cases of human breast cancer and has been shown to promote cancer cell migration in carcinoma cells of the breast, prostate gland and thyroid gland. In oestrogen receptor alpha-negative MDA-MB-231 human breast cancer cells, relaxin was shown to down-regulate the metastasis-promoting protein S100A4 (metastasin), a highly significant prognostic factor for poor survival in breast cancer patients. The cellular mechanisms of relaxin exposure in breast cancer cells are not fully understood. The aim of this study was to investigate short-term and long-term effects of relaxin on cancer cell motility and S100A4 expression and to determine the long-term effects of relaxin on *in vivo *tumour growth in an oestrogen-independent context.

**Method:**

We have established stable transfectants of highly invasive oestrogen-receptor alpha-negative MDA-MB-231 human breast cancer cells with constitutive expression of bioactive H2-relaxin (MDA/RLN2). RLN2 secretion was determined by ELISA. Relaxin receptor RXFP1 (Relaxin-family-peptide) was detected by reverse transcription (RT) PCR and its activation was assessed by induction of cyclic adenosine monophosphate (cAMP). Stable MDA/RLN2 clones and RLN2 treated MDA-MB-231 cells were subjected to motility and *in vitro*-invasion assays. Proliferation was assessed in bromodeoxyuridine (BrdU) and MTT assays. S100A4 expression was determined by RT-PCR and Western blot. Specific small interfering RNA was employed to down-regulate relaxin receptor and S100A4. MDA/EGFP vector control and two MDA/RLN2 clones were injected subcutaneously in nude mice to determine tumour growth and cancer cell invasiveness *in vivo*. Xenograft tumour tissues were assessed by histology and immunohistochemistry and frozen tissues were used for the detection of S100A4 and RLN2.

**Results:**

Short-term exposure to relaxin for 24 hours increased cell motility in a relaxin receptor-dependent manner. This increase in cell motility was mediated by S100A4. Long-term exposure to relaxin secreted from stable transfectants reduced cell motility and *in vitro *invasiveness. Relaxin decreased cell proliferation and down-regulated cellular S100A4 levels in MDA-MB-231 and T47D breast cancer cells. Stable MDA/RLN2 transfectants produced smaller xenograft tumours containing reduced S100A4 protein levels *in vivo*.

**Conclusion:**

Our results indicate that long-term exposure to relaxin confers growth inhibitory and anti-invasive properties in oestrogen-independent tumours *in vivo*, which may in part be mediated through a down-regulation of S100A4.

## Introduction

The polypeptide hormone relaxin is increased in human breast carcinoma tissues [1]. In all human breast tumours investigated, immunoreactive H2 relaxin (RLN2) was localised to the cytoplasm of neoplastic epithelial cells [1]. The expression of RLN1 and RLN2 genes in neoplastic breast tissues demonstrated the local production of relaxin in breast carcinoma [1]. Relaxin was also shown to be increased in human carcinoma of the prostate gland [2], endometrium [3] and thyroid gland [4,5].

In addition, the breast is a physiological producer and target for relaxin and mammary epithelial cells, and myoepithelial cells in normal human breast tissue of prepubertal and all ages of adult women were shown to produce relaxin [1,6,7]. Apart from placental production during pregnancy [8,9], relaxin was not detectable in the plasma of cycling and lactating women suggesting local production and autocrine/paracrine actions of relaxin within the glandular breast tissue. The recently reported elevated relaxin serum levels in women with metastatic breast cancer suggest that relaxin may be a novel marker associated with breast cancer metastasis [10]. Relaxin binds predominantly to the 7-transmembrane G-protein-coupled receptor LGR7 [11-13], also known as the relaxin/insulin-like family peptide receptor 1 (RXFP1) [14]. Activation of RXFP1 by relaxin caused elevation of cyclic adenosine monophosphate (cAMP) levels [15,16]. *In vitro *studies have linked relaxin expression with invasive behaviour of cancer cells [4,17-19]. With treatment with porcine relaxin, SK-BR3 and MCF-7 human breast cancer cells showed increased secretion of MMP-2, MMP-9 and MMP-7 causing increased migration through matrigel [19]. Adenoviral-mediated expression of human prorelaxin in CF33.MT canine mammary carcinoma cells was described to enhance *in vitro *invasiveness through a laminin matrix [20]. Whether these pro-migratory effects of relaxin were mediated by RXFP1 is unknown. Earlier studies have also reported that porcine relaxin reduces proliferation and induces differentiation in the oestrogen receptor-alpha (ERα)-positive breast cancer cell line MCF-7 *in vitro *[21,22] and *in vivo *[23]. Until now, these conflicting actions of relaxin in breast cancer cells have not been fully understood. Although *in vivo *studies in rat uterus have provided evidence for a crosstalk between RXFP1 and ERα and demonstrated ERα-mediated oestrogenic effects with relaxin treatment [24,25], a direct oestrogenic action of relaxin in breast cancer has not been described. Oestrogen-independent breast cancer usually shows a more aggressive clinical course and poorer treatment responses. Currently, little information is available on the role of relaxin in ERα-negative human breast cancer cells.

We have previously identified the EF-hand (helix-turn-helix structural domain) calcium-binding protein S100A4 (metastasin) as a relaxin target in oestrogen-independent, ERα-negative, highly invasive MDA-MB-231 human breast cancer cells [26]. S100A4 was linked to cancer cell invasiveness and metastasis in various human cancer types [27-31] and *in vivo *cancer models [32-36]. Expression of the S100A4 protein is positively correlated with breast cancer invasiveness of rodent mammary carcinoma cells [37] and serves as an independent prognostic marker that is highly significant for poor survival in breast cancer patients [29]. Both, extracellular and intracellular functions of S100A4 contribute to tissue invasiveness. The interaction with non-muscle myosin heavy chain IIA has been extensively studied [38] and was associated with migratory behaviour [39-41]. Extracellular S100A4 was also shown to stimulate angiogenesis [32].

ERα-negative human breast cancers carry a high risk of metastatic disease and have poor therapeutic responses [42,43]. The aim of the present study was to investigate the effect of long-term relaxin exposure – mimicking the persistent exposure to relaxin produced by breast carcinoma cells within the cancer tissue – on *in vitro *cell migration and *in vivo *tumour growth in an oestrogen-independent breast cancer cell model. We propose a novel causal relationship between relaxin expression, attenuated expression of S100A4 and reduced tumour growth.

## Materials and methods

### Cell culture and relaxin exposure

MDA-MB-231 and T47D human breast cancer cells were cultivated in HAM's F12 medium (Biochrom, Berlin, Germany) substituted with 10% fetal calf serum (FCS), 2 mmol L-glutamine (Gibco, Invitrogen, Burlington, ON, Canada), 6 ng/ml insulin (Gibco; Invitrogen Corp, Burlington, ON, Canada), 3.75 ng/ml hydrocortisone (Sigma, Oakville, ON, Canada). Stable MDA-MB-231/pIRES-EGFP-RLN2 and MDA-MB-231/pIRES-EGFP vector control transfectants, referred to as MDA/RLN2 and MDA/EGFP, respectively, were cultured in the same medium supplemented with 800 μg/ml of the selective antibiotic Geneticin (Gibco; Invitrogen Corp, Burlington, ON, Canada). MCF7 cells were propagated in MEM with Earle's salts (Biochrom AG, Berlin, Germany) supplemented with 10% FCS, 2 mmol L-glutamine, 1 nM sodium pyruvate (Gibco, Invitrogen Corp, Burlington, ON, Canada) and 0.8 μg/ml insulin (Gibco, Invitrogen). All cells were incubated in a 5% carbon dioxide atmosphere at 37°C. The culture medium was changed every second day and cells were passaged every two to four days using accutase (PAA Laboratories GmbH, Linz, Austria). Cells were frozen in 10% DMSO (dimethyl sulfoxide) containing culture medium and stored in liquid nitrogen. Before treatment with 100 ng/ml and 500 ng/ml RLN2 (Phoenix Pharmaceuticals, Belmont, USA) in 1% FCS culture medium, cells were adjusted to 1% FCS-containing culture medium for 24 hours. For incubation periods longer than 24 hours, medium with fresh RLN2 at the same concentration was changed daily.

### Stable MDA-MB-231-pIRES transfectants

The generation of stable transfectants MDA/RLN2 and MDA/EGFP was described previously [26]. A pre-proRLN2 pIRES-EGFP construct was used and five MDA/RLN2 and two MDA/EGFP clones were employed for this study.

### Transcriptional analysis

Total RNA of the stable MDA/RLN2 and MDA/EGFP transfectants was extracted using the Trizol reagent (Life Technologies, Karlsruhe, Germany) and the amount of total RNA was determined by spectrophotometry at 260 nm/280 nm (Ultrospec 3300 pro, Amersham, USA). Extraction of total RNA from nude mouse tumour tissues was performed with the Trizol reagent after homogenisation of the frozen tissue samples. Total RNA (1 μg each) was used for the preparation of first strand cDNA employing the superscript reverse transcriptase II (Gibco, Berlin, Germany) and 100 ng/μl of random primer 50 (Invitrogen, Burlington, ON, Canada). For the amplification of pre-proRLN2 and the partial coding sequence of human RXFP1 intron-spanning oligonucleotide primers (Table 1) were employed to preclude any genomic DNA amplification. For semi-quantitative reverse transcriptase (RT) PCR analysis, the 18S amplification in each cDNA preparation served as a reference in determining relative cDNA levels per sample using the BIO 1D software (LTF, Wasserburg, Germany). The following conditions were used: 2 μl of cDNA, 2.5 μl of 10× Advantage2 polymerase mix buffer (Clontech, Mountain View, CA, USA), 100 μM of dNTP, 10 pmol of each primer (Table 1) and 2 U Taq DNA-polymerase (Life Technologies, Invitrogen, Canada) in a final volume of 25 μl. The amplification conditions were optimised for each gene and consisted of 35 PCR cycles for the respective target genes, with the exception of 18s mRNA detection which was performed for 10 cycles (Table 1). PCR cycles consisted of an initial denaturation for three minutes at 95°C, followed by several cycles of denaturation at 95°C (Table 1), annealing temperature specific for each primer (Table 1), both for one minute each, an elongation step for two minutes at 72°C and a final extension cycle for 10 minutes at 72°C. Amplicons were separated on a 2% agarose gel. Kodak software (Kodak Digital Science 1D v.3.0.2.; Kodak, Toronto, ON, Canada) was used to perform semi-quantitative assessment of the specific PCR-products by densitometry using the 18S amplicons as cDNA-specific internal standard.

**Table 1:** Oligonucleotide primers employed at melting temperatures (T_M _in °C) used to determine the expression of RLN2, human RXFP1 relaxin receptor (RXFP1), S100A4 and 18S transcripts in human breast cancer cell lines and xenograft tumours.

**Primer**	**size**	**primer sequence (5'-3')**	**T_M_**	**cycles**
S100A4	289 bp	sense – gaa ggc cct gga tgt gat ggt g	65°C	35
		antisense – cat ttc ttc ctg ggc tgc tta tc		
RLN2	485 bp	sense – tct gtt tac tac tga acc aat tt	55°C	35
		antisense – cat ggc aac att tat tag cca a		
RXFP1	243 bp	sense – ccc aat tct cta tac tct gac cac aag	65°C	35
		antisense – tca tga ata gga att gag tct cgt tga tt		
18S	344 bp	sense – gtt ggt gga gcg att tgt ctg g	62°C	10
		antisense – agg gca ggg act taa tca acg c		

### Relaxin ELISA

MDA/RLN2 and MDA/EGFP transfectants were seeded at 1 × 10^5 ^cells/ml per well and incubated for 24 hours at 37°C in a humidified carbon dioxide incubator. The cell culture supernatant was collected and the concentration of secreted relaxin was analysed by ELISA according to the manufacturer's protocol (ImmunoDiagnostic, Bensheim, Germany). The colour reaction was measured at 450 nm with an ELISA reader (SLT Labinstruments GmbH, Crailsheim, Germany).

### cAMP assay

Intracellular cAMP levels were measured in MDA-MB-231 breast cancer cells. Cells were seeded at 1 × 10^4 ^cells per well in 96 well plates. Before treatment, cells were incubated with 1 mM 3-isobutyl-1-methyl-xanthine ([IBMX] Sigma, Steinheim, Germany) for two hours at 37°C. MDA-MB-231 cells were then incubated with 100 ng/ml recombinant human RLN2 or 100 μl/well of the supernatants of the MDA/RLN2 and MDA/EGFP transfectants, respectively, for 30 minutes at 37°C in the presence of 1 mM IBMX. These supernatants had been collected from the stable transfectants (1 × 10^5 ^cells/well) cultured overnight in six-well plates and centrifuged at 3000 × g for 10 minutes at 4°C to remove remaining cells. Treatment with 10 μM Forskolin (Calbiochem-Novabiochem Corp., San Diego, CA, USA) served as positive control for the induction of cellular cAMP. Cells were harvested, washed and lysed with cAMP extraction buffer and cAMP levels were detected with the colourimetric cAMP Biotrak enzyme immunoassay according to the manufacturer's instructions (EIA, Amersham, Freiburg, Germany). The reaction was stopped in 1 M H_2_SO_4 _and the product was measured at 450 nm in a microplate ELISA reader (SLT Laboratory Instruments, Gröding, Austria).

### Bromodeoxyuridine assay

Cellular proliferation was determined using a competitive colourimetric cell proliferation ELISA assay (Roche Diagnostics, Mannheim, Germany) as described previously [4]. Briefly, MDA/RLN2 and MDA/EGFP transfectants were seeded at 5 × 10^3 ^cells per 96 well in 100 μl culture medium and cultured for 24 hours before adding 10 μl bromodeoxyuridine (BrdU) labelling solution for seven hours. The assay was performed according to the manufacturer's instructions. The absorbance was measured within five minutes in an ELISA reader (SLT Laboratory Instruments, Gröding, Austria) at 450 nm/620 nm.

### Cell cytotoxicity (MTT) assay

For cytotoxicity measurement, the NADH_2_-dependent cell viability assay kit (Easy-for-you; Biomedica, Wien, Austria) was used as previously described [4].

### Luminometric ATP assay

Stable transfectants plated in 96 well plates at a density of 0.25, 0.5 and 1 × 10^4 ^cells/well were used as previously described [4].

### Motility assay

Cellular motility was evaluated in 24-well Transwell chambers (Techno Plastic Products AG, Trasdingen, Switzerland) as previously described [4]. Briefly, MDA-transfectants (1 × 10^4 ^cells) were plated on a polycarbonate filter insert with a pore size of 8 μm (Thin Certs™-TC Inserts, Greiner bio-one, Germany) in 200 μl culture medium with 1% FCS (upper chamber). The lower chamber was filled with 500 μl culture medium containing 1% FCS. After 24 hours, filter inserts were removed and cells remaining on top of the filter were wiped off with cotton swabs. Cells migrated through the membrane pores to the underside of the membrane and were washed with phosphate buffered saline (PBS), fixed in PBS/methanol (Merck, Darmstadt, Germany) and stained with 0.1% toluidine blue (Merck, Darmstadt, Germany) in 2.5% sodium carbonate (Roth, Karlsruhe, Germany). Cells were counted under the light microscope (Zeiss, Jena, Germany) in five separate high-power fields per filter.

To determine the short-term paracrine effects of RLN2, MDA-MB-231 cells were seeded on top of the filter and recombinant human RLN2 (rhRLN2, Phoenix, Burlingame, CA, USA) was added at 100 ng/ml or 500 ng/ml to the upper and lower chamber. To determine the paracrine effect of RLN2 secreted by MDA/RLN2 stable transfectants on MDA-MB-231 cell motility, MDA/RLN2 and MDA/EGFP (control) were plated in the bottom chamber at 5 × 10^4 ^cells in 600 μl culture medium with 1% FCS and cultured overnight at 37°C. MDA-MB-231 cells seeded at 1 × 10^4 ^cells on the top of the filter were then inserted into the 24 wells containing the transfectants in the lower chamber. After incubation for 24 hours, filters were processed and migrated cells counted as described above.

To determine the long-term effects of rhRLN2 on cell motility, MDA-MB-231 were incubated for seven days with 500 ng/ml rhRLN2 before seeding on top of the filter with culture medium containing 1% FCS and 500 ng/ml rhRLN2. Migrated cells were counted after 24 hours. All experiments described here were performed at least in triplicate and were expressed as mean ± SEM.

### *In vitro *invasion assay

For the assessment of the invasive abilities of MDA/RLN2 transfectants a modified 24-well Transwell assay was used as previously described [4]. Briefly, polycarbonate, translucent filters with a pore size of 8 μm were coated with 1 μg/cm^2 ^to 2 μg/cm^2 ^laminin (from human placenta, Sigma, Oakville, ON, Canada) and 0.8 mg/ml collagen A (from placenta, Biochrom KG, Cambridge, UK). Cells were seeded on top of the coated filters and the experiment was performed as described for the motility assay. All experiments were performed at least in triplicate and were expressed as mean ± SEM.

### siRNA knock-down experiments for RXFP1 and S100A4

Transcriptional knock-down was achieved by transient transfections of MDA-MB-231 cells with specific small interfering (si) RNAs to human RXFP1 (sense-r [GCAGUUACCUGCUUUGGAA]dTdT and antisense-r [UUCCAAAGCAGGUAACUGC]dAdG; Qiagen, Germany), human S100A4 (sense-GGGUGACAAGUUCAAGCUCtt and antisense-GAGCUUGAACUUGUCACCCtc; Ambion, Cambridgeshire, UK) and human negative control siRNA (sense-UUCUCCGAACGUGUCACGUdt dt and antisense-ACG UGACACGUUCGGAGAAdt dt, Qiagen, Germany). The siRNA at 150 nM (RXFP1 and negative control siRNA) and 200 nM (S100A4 and negative control siRNA) was complexed with Lipofectamine-2000 (Invitrogen, Burlington, ON, Canada) in serum-free OptiMem medium (Gibco, Invitrogen Corp, Burlington, ON, Canada). MDA-MB-231 cells (1 × 10^5 ^cells) were seeded at 60% confluency in six-well plates and incubated with the transfection mix for 24 hours at 37°C. The transfection mix was replaced with normal medium. Cells transfected with non-silencing siRNA and cells incubated with Lipofectamine-2000 alone served as negative controls. Cells were used in motility assays 48 hours after siRNA transfections. Total RNA was extracted with Trizol one, two and three days after transfections.

### Western blot analysis

For S100A4 protein extraction, cells were harvested, washed with PBS (Ca^2+^-/Mg^2+^-free Biochrom AG, Germany) and lysed with two times Laemmli extraction buffer under reducing conditions [125 mmol/L Tris-HCl, pH 6.8, 4% SDS, 20% glycerol, 10% mercaptoethanol (ME); 2% bromophenol blue; all reagents from Sigma] containing a protease inhibitor cocktail (Sigma, Oakville, ON, Canada). On a 15% SDS gel, 40 μl protein extracts were separated. After blotting onto PVDF membranes (GE Healthcare, Buckinghamshire, UK) nonspecific binding was blocked in PBS containing 0.1% Tween (PBS-T) (EMD, San Diego, USA) and 3% BSA (Roth GmbH & Co, Karlsruhe, Germany) for two hours at room temperature. A rabbit polyclonal antibody to S100A4 (Ab-8; Dunn Labortechnik, Asbach, Germany) was diluted 1:500 in blocking buffer and incubated overnight at 4°C. After thorough washing with PBS-T a HRP (horseradish peroxidase)-conjugated secondary goat anti-rabbit antibody (Dianova, Hamburg, Germany) at 1:20,000 was employed for one hour at room temperature. After washing with PBS-T, the proteins were visualised with the ECL kit (Amersham, Piscataway, NJ, USA).

### Tumour study in nude mice

MDA/EGFP and MDA/RLN2 (clones 19 and 23) at 2 × 10^7 ^cells/ml in medium without FCS were subcutaneously injected into the abdominal region on both sides of each mouse. Male nude mice (NMRI) of three weeks of age were used. This study was approved by the animal ethics committee of the Martin Luther University, Faculty of Medicine. Five mice were injected per transfectant resulting in a maximum of 10 tumours to be analysed for each transfectant. Tumour growth was measured twice weekly. Primary tumour volumes were calculated according to the mathematical formula for ellipsoid shapes. After 60 days the mice were euthanased. Primary tumours were fixed in 4% paraformaldehyde and embedded in paraffin for histological examination and cryopreserved for RNA and protein extraction.

### Histological staining and immunohistochemistry

Paraformaldehyde-fixed and paraffin-embedded tumour tissues were stained with haematoxylin and eosin for histological analysis. Picrosirius red stain was performed according to the method previously described [44]. For the detection of immunoreactive smooth muscle actin, a mouse monoclonal antibody (A5228, Sigma-Aldrich, Oakville, ON, Canada) was used. Briefly, dewaxed 5 μm sections were equilibrated in PBS-T. Endogenous peroxidase activity was inactivated with 3% hydrogen peroxide in methanol for 20 minutes at room temperature. Nonspecific binding sites were blocked with PBS-T containing 10% goat non-immune serum. Tissue sections were incubated overnight at 4°C with the mouse anti-actin antibody diluted 1:500 in blocking buffer. After washing, sections were incubated for one hour at room temperature with a peroxidase-conjugated goat anti-mouse secondary antibody (Dianova, Hamburg, Germany) diluted 1:500 in PBS-T. Specific binding was visualised with the peroxidase substrate diaminobenzidine ([DAB] Pierce, Rockford, IL). Isotype control mouse IgG (Sigma) at the same dilution served as a negative control. For Ki67 staining, a rabbit monoclonal antibody (SP6, Abcam, Cambridge, MA, USA) was used according to the manufacturer's instructions using an alkaline phosphatase detection method.

For the immunodetection of RXFP1 in MDA-transfectants, cells were grown on coverslips and fixed in 4% paraformaldehyde. Sections were boiled in citrate buffer, blocked with 1% BSA and 3% non-immune goat serum and incubated overnight with a rabbit polyclonal antibody to RXFP1 (Phoenix Pharmaceuticals, Burlingame, CA, USA) diluted 1:200 in blocking buffer. A biotinylated goat anti-rabbit secondary antibody was applied (BA-1000, Vector Laboratories, Burlington, ON, Canada) at 1:200 and specific staining was detected with the ABC detection kit (Vector Laboratories, Burlington, ON, Canada). Rabbit non-immune serum was used as negative control.

### TUNEL assay

A TUNEL (dUTP nick end labelling) assay for the detection of DNA fragmentation in apoptotic nuclei was performed on the paraffin sections of six MDA/EGFP and six MDA/RLN2 nude mouse tumours using the TACS.XL In situ Apoptosis Detection Kit (Trevigen Inc, Gaithersburg, MD, USA) according to the manufacturer's instructions.

## Results

### Short-term exposure to relaxin causes increased cell motility in MDA-MB-231 human breast cancer cells and is dependent on RXFP1-signalling

**Figure 1:**
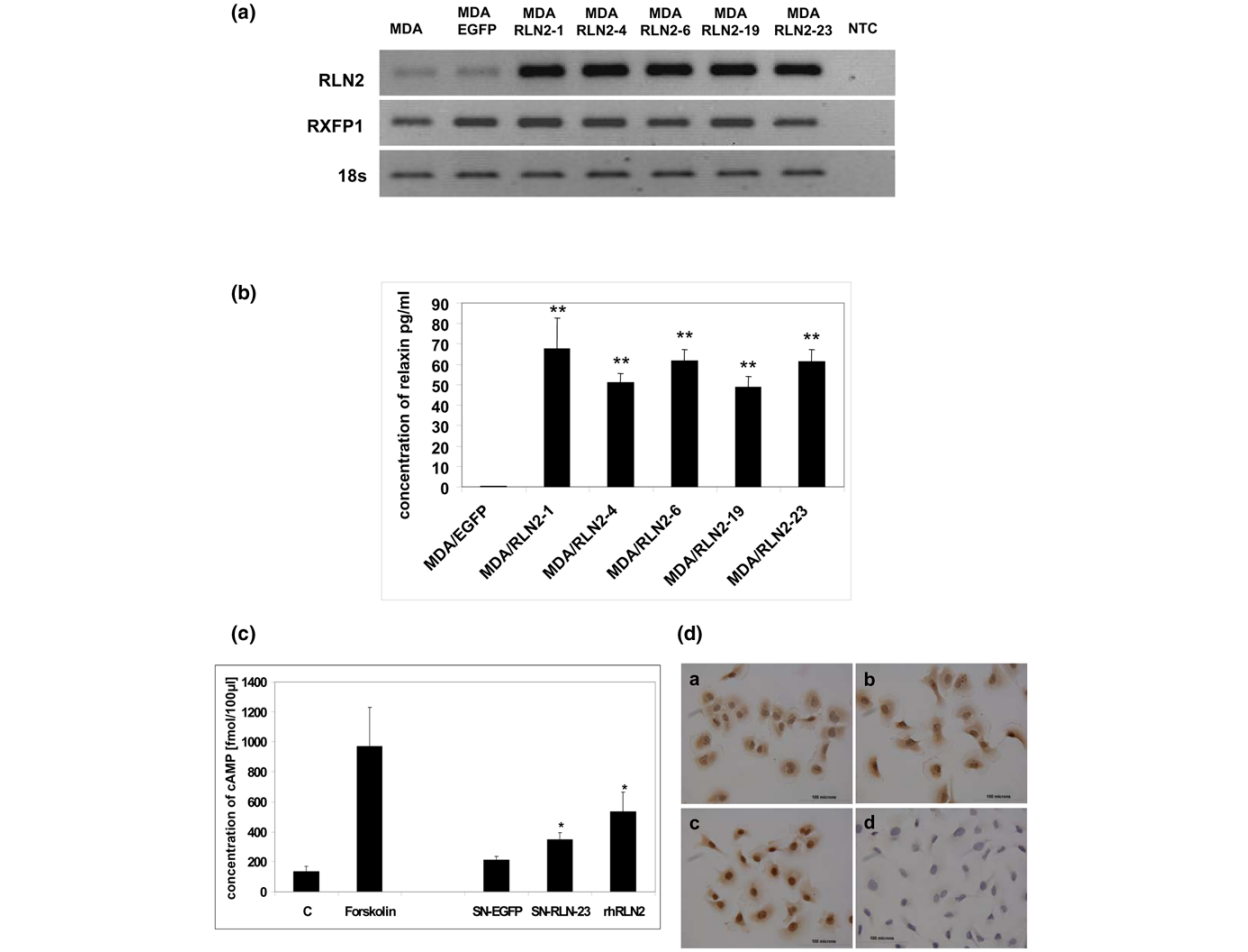
**Stable MDA/RLN2 transfectants secrete bioactive RLN2 and express functional RXFP1**. (a) Reverse transcriptase polymerase chain reaction (RT-PCR) analysis was performed showing increased expression of RLN2 transcripts in all five MDA/RLN2 transfectants when compared with MDA/EGFP-vector controls. Employing intron spanning primers encoding for the relaxin receptor RXFP1, a single transcript encoding RXFP1 was detected in both MDA/EGFP and MDA/RLN transfectants. 18S transcript levels were used as reference. (b) Secreted relaxin was quantified in the culture medium using a human relaxin-ELISA. MDA/RLN2 transfectants secreted significantly higher amounts of RLN2 compared with the MDA/EGFP control; ** p ≤ 0.005. (c) A cAMPassay was used to determine the bioactivity of secreted RLN2. Intracellular cAMP concentration was determined in MDA-MB-231 cells following incubation with 100 ng/ml rhRLN, the culture supernatants of MDA/RLN2 clone 23 (SN-RLN-23) or of MDA/EGFP (SN-EGFP) transfectants. Forskolin (10 μM) was used as positive control and untreated cells served as negative control. Induction of cAMP by secreted RLN2 and rhRLN2 demonstrates the presence of a functional RXFP1 receptor system in this cell line. Experiments were performed in triplicates; data were presented as mean ± SEM. Statistical significance was determined with student t-test and p ≤ 0.05 were marked with an asterisk. (d) The presence of RXFP1 protein in stable MDA/EGFP and MDA/RLN2 transfectants was detected by immunocytochemistry using a rabbit polyclonal antibody to human RXFP1. Positive staining for RXFP1 was obtained for all cell clones and shown here for (ai) MDA/EGFP, (bi) MDA/RLN2-4 and (ci) MDA/RLN2-19. (di) A negative control using a rabbit non-immune serum did not result in any staining.

Stable transfectants of the human ERα-negative breast cancer cell line MDA-MB-231 over-expressing human RLN2 (MDA/RLN2) were described previously to express increased amounts of proRLN2 [26]. MDA/RLN2 transfectants expressed transcripts for RLN2 and the relaxin receptor RXFP1 (Figure 1a). Using a human relaxin-ELISA, these MDA/RLN2 transfectants were shown to secrete more than 50 times the amount of RLN2 when compared with MDA/EGFP controls (Figure 1b). The presence of RXFP1 protein in stable MDA/EGFP and MDA/RLN2 transfectants was demonstrated by immunohistochemistry (Figure 1d). Functionality of RXFP1 in MDA-MB-231 human breast cancer cells was determined by the increase in intracellular cAMP production following exposure to recombinant human RLN2 (rhRLN2; Figure 1c). When exposed to the supernatants of MDA/RLN2 and MDA/EGFP vector control clones, only the supernatant of MDA/RLN2 containing secreted relaxin caused an increase in cAMP production in MDA-MB-231 cells showing the secreted proRLN2 to be bioactive (Figure 1c).

**Figure 2:**
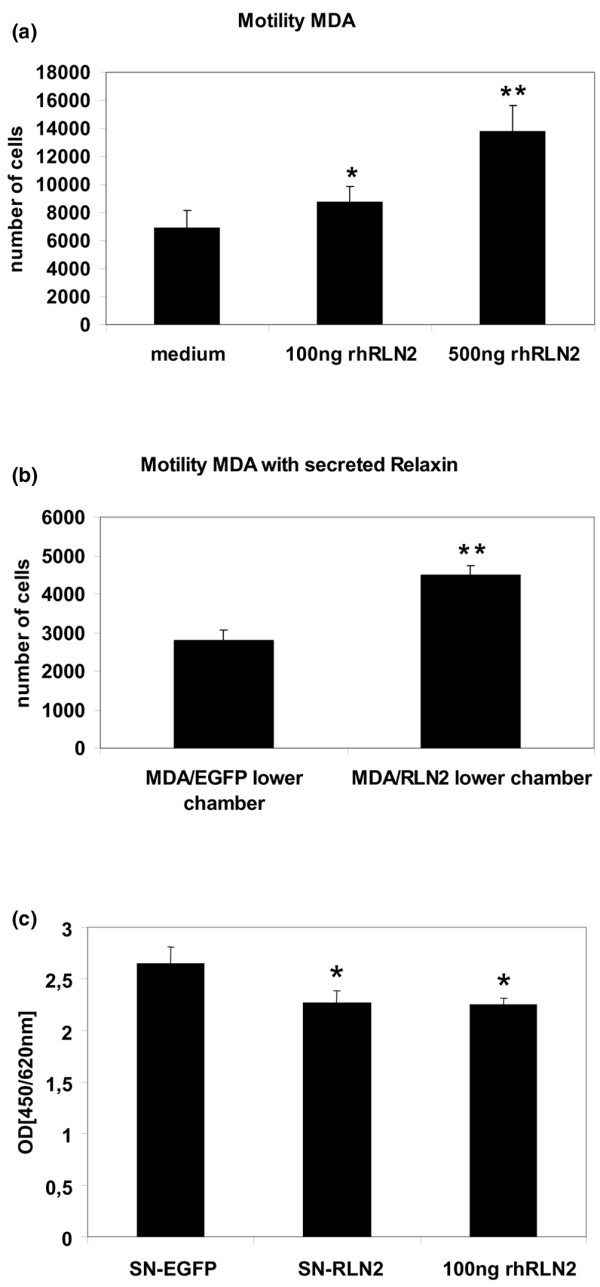
**Short-term exposure to exogenous RLN2 increases cell motility dose-dependently**. Relaxin-induced changes in MDA-MB-231 cellular motility were determined after 24-hour exposure using an 8 μm porous membrane migration assay. Cells which migrated through the 8 μm pores to the underside of the membrane were stained, five microscopic fields were counted for each filter and the results of three independent experiments are shown in the graph as mean ± SEM for (a) MDA-MB-231 after incubation with 100 ng/ml and 500 ng/ml recombinant human (rh) RLN2 and (b) after exposure to secreted RLN2 from stable transfectants seeded in the lower chamber, shown here for MDA/EGFP and MDA/RLN2 clone 23. To assess proliferation, (c) MDA-MB-231 cells were treated with the supernatant of MDA/EGFP control transfectants (SN-EGFP) and of MDA/RLN2 transfectants (SN-RLN2) or with rhRLN2 at 100 ng/ml for 24 hours and proliferation was determined with a bromodeoxyuridine (BrdU) assay. rhRLN2 and secreted RLN2 slightly, but significantly reduced cell proliferation in MDA-MB-231 cells. Experiments were performed in triplicates and data were presented as mean ± SEM. Statistical significance was assessed by (a) one-way ANOVA and with (b, c) student t-test using the SPSS software package (SPSS GmbH, München, Germany). *p < 0.05; **p ≤ 0.005.

Two-chamber motility assays were performed to investigate changes in cell motility with short-term relaxin treatment in MDA-MB-231. A 24 hour exposure to 100 ng/ml and 500 ng/ml rhRLN2 increased cell motility in MDA-MB-231 in a dose-dependent manner (Figure 2a). When MDA-MB-231 cells were exposed to the culture supernatants of stable MDA/RLN2 and MDA/EGFP transfectants, only MDA-MB-231 cells exposed to the supernatants of MDA/RLN2 transfectants showed an increase in cell motility (Figure 2b) demonstrating that secreted RLN2 also induced cell motility after 24 hours. To exclude a relaxin-induced increase in cell proliferation as being responsible for the increased number of migrated cells, a BrdU assay was performed on MDA-MB-231 cells after 24 hour exposure to rhRLN2 and to the secreted RLN2 contained in the supernatants of MDA/RLN2 clones. Our results demonstrated that relaxin did not act as a growth factor in MDA-MB-231 cells but, instead, caused reduced cell proliferation (Figure 2c).

**Figure 3:**
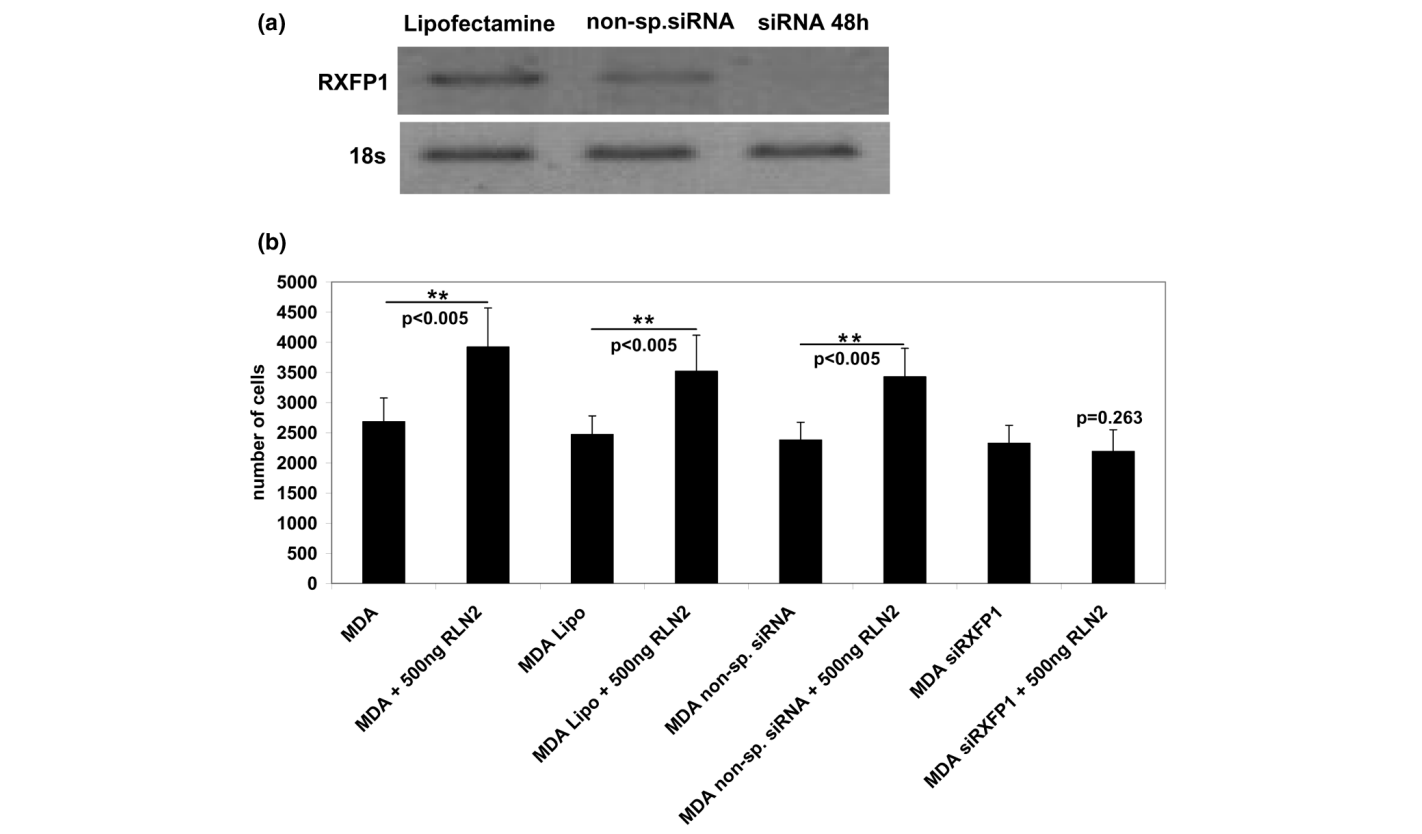
**Relaxin's effect on motility is RXFP1-mediated**. RXFP1 relaxin receptor was down-regulated with 150 nM siRNA against RXFP1 (LGR7) in MDA-MB-231 cells. Cells treated with the vehicle Lipofectamine 2000 only or transfected with 150 nM non-silencing small interfering (si) RNA were used as controls. (a) Semi-quantitative PCR analysis was performed with specific primers for RXFP1, shown here for 48 hours after transfection. (b) Motility of MDA-MB-231 cells seeded at 1 × 10^4 ^cells was assessed after RXFP1 knock-down with RXFP1 siRNA. The results of three independent experiments are shown in the graph as mean ± SEM, a two-way ANOVA analysis was performed with the SPSS software package and a p < 0.05 was considered significant at the 95% confidence level as shown by the asterisks (**).

The short-term increase in MDA-MB-231 cell motility in response to RLN2 was mediated by the RXFP1 as determined by specific siRNA knock-down experiments. Transient transfections with a RXFP1 siRNA probe resulted in a significant reduction of RXFP1 transcript levels in MDA-MB-231 (Figure 3a) and siRNA RXFP1-treated MDA-MB-231 failed to respond with increased motility after 24 hours of incubation with rhRLN2 (Figure 3b). In contrast, MDA-MB-231 cells exposed to lipofectamine or transfected with a non-silencing siRNA probe responded to rhRLN2 with increased motility (Figure 3b).

### Long-term exposure to relaxin decreases cell motility and in vitro invasiveness in MDA-MB-231 human breast cancer cells

**Figure 4:**
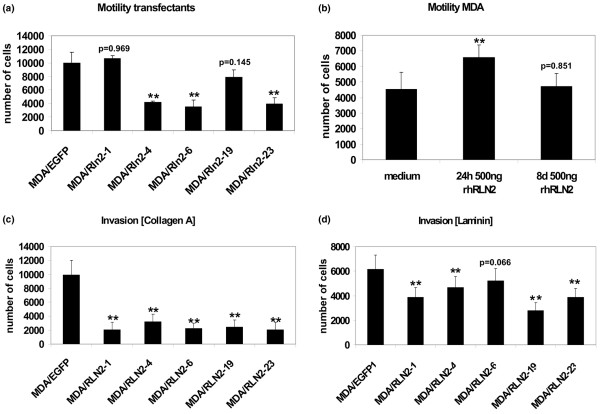
**Long-term exposure to relaxin decreases cell motility and *in vitro *invasiveness**. (a) MDA/RLN2 and MDA/EGFP transfectants were subjected to motility assays as described for Figure 2. (b) In contrast to a 24-hour short-term exposure, long-term incubation of MDA-MB-231 cells with 500 ng/ml rhRLN for eight days did not cause changes in cellular motility with the number of migrated cells remaining unchanged when compared with controls (medium). Experiments were performed in triplicates and five microscopic fields were counted for each filter. The results are shown in the graph as mean ± SEM. (a, b) One-way ANOVA analysis was performed. **p < 0.005 when different from (a) MDA/EGFP or (b) medium treatment. MDA/RLN2 and MDA/EGFP transfectants were seeded on 8 μm porous filters coated with (c) collagen A and (d) laminin to assess *in vitro *invasiveness. Experiments were assessed as described for motility assays. The results of three independent experiments are shown in the graph as mean ± SEM. One-way ANOVA analysis was performed. **p < 0.005 when different from MDA/EGFP control.

To investigate whether long-term exposure to relaxin influenced cell migration in MDA-MB-231 cells, we employed the stable MDA/RLN2 and MDA/EGFP clones in cell motility and migration assays. Four out of five MDA/RLN2 transfectants with constitutive expression of RLN2 showed a reduction in cell motility when compared with the MDA/EGFP vector controls (Figure 4a). To determine whether the longer exposure time to relaxin caused the observed decrease in cell motility, we performed motility assays on MDA-MB-231 cells after long-term treatment with rhRLN2 for seven days. Motility was assessed following an additional 24 hour exposure to rhRLN2. In contrast to MDA-MB-231 cells exposed to rhRLN2 for 24 hour only, cells treated with rhRLN2 for seven days were unable to increase cell motility in response to an additional 24 hour exposure to rhRLN2 (Figure 4b). Thus, the increase in cellular motility observed in untransfected MDA-MB-231 was a temporary phenomenon during the first 24 hours of exposure to relaxin and was not observed with long-term relaxin incubations and in MDA/RLN2 transfectants constitutively producing relaxin. To determine whether relaxin altered the invasive potential in MDA-MB-231, cell migration through components of the extracellular matrix was tested. MDA/RLN2 transfectants showed a reduced ability to invade through collagen A (Figure 4c) and laminin (Figure 4d) when compared with the MDA/EGFP vector controls.

**Figure 5:**
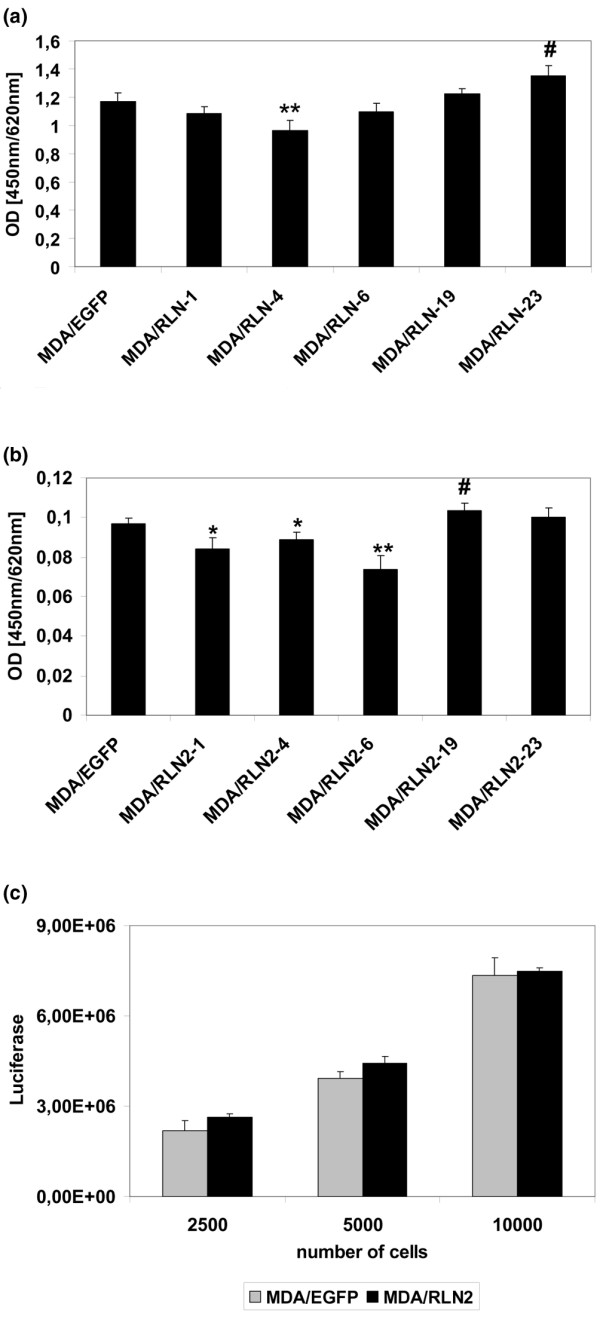
**Long-term exposure to relaxin does not stimulate proliferation in MDA-MB-231**. Proliferation of MDA/EGFP and MDA/RLN2 transfectants was determined with (a) bromodeoxyuridine (BrdU) and (b) NADPH_2_-dependent MTT proliferation assays. Experiments were performed in triplicates and shown in the graph as mean ± SEM. The t-test features of the SPSS 10.0 software were employed to calculate for statistical significance of MDA/RLN2 compared with MDA/EGFP. p < 0.05 was marked in the graph as reduction (*) or as increase (#). **p < 0.005. (c) A luminometric ATP assay was performed to assess the metabolic activity and no changes were determined in MDA/RLN2 compared with MDA/EGFP clones.

To investigate whether a reduction in cell proliferation played a role in the long-term effects of relaxin on MDA-MB-231 cells, we performed BrdU and MTT proliferation assays on MDA/RLN2 transfectants. Cell proliferation was reduced in one out of five and three out of five MDA/RLN2 clones when tested in a BrdU or MTT assay, respectively (Figures 5a, b) A luminometric ATP assay was applied to MDA/RLN2 transfectants and did not show any changes in metabolic activity compared with MDA/EGFP vector controls (Figure 5c).

### S100A4 is down-regulated by relaxin in breast cancer cells and S100A4 siRNA knock-down prevents relaxin-induced increase in motility

**Figure 6:**
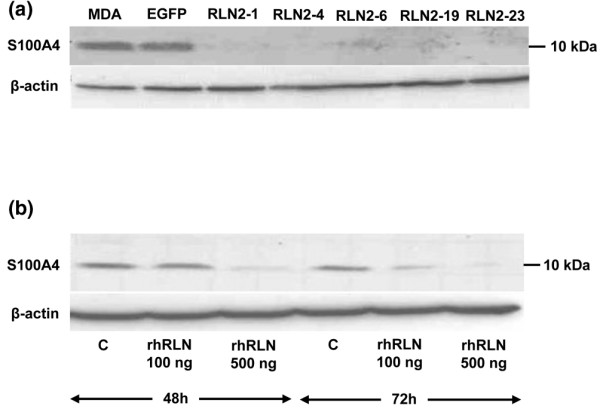
**S100A4 protein is down-regulated by relaxin**. The 10 kDa S100A4 protein was detected by Western blot analysis in cellular extracts of MDA-MB-231 cells, MDA/EGFP and MDA/RLN2 transfectants. (a) All MDA/RLN2 transfectants contained negligible levels of S100A4 protein when compared with MDA-MB-231 or MDA/EGFP vector controls. (b) In T47D cells, S100A4 protein was also down-regulated following 48 hour and 72 hour exposure to 100 ng/ml or 500 ng/ml rhRLN2. Beta-actin detection was used to control for equal protein loading of the gels.

**Figure 7:**
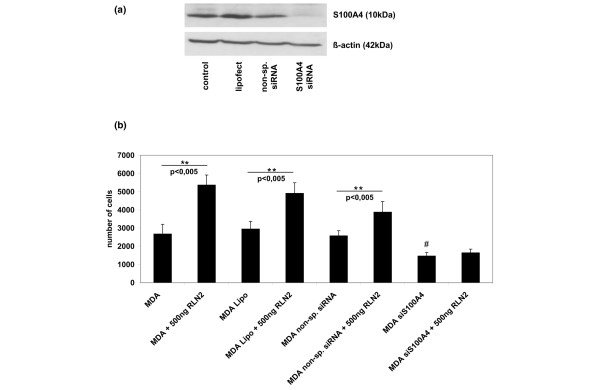
**S100A4 siRNA knock-down abolishes relaxin's effect on cell motility**. Treatment of MDA-MB-231 cells with 200 nM S100A4 siRNA, but not with 200 nM non-silencing small interfering (si) RNA or Lipofectamine, resulted in down-regulation of S100A4 protein after 48 hours and 72 hours as shown here (a) after 48 hours. (b) When S100A4 siRNA treated MDA-MB-231 cells were subjected to 24 hours motility assays, relaxin was unable to increase cell motility. In contrast, cells transfected with non-silencing siRNA probe and cells treated with lipofectamine only were able to respond to 500 ng/ml rhRLN2 with an increase in motility similar to untreated cells. The results of three independent experiments are shown in the graph as mean ± SEM. One-way ANOVA analysis was performed. **p < 0.005 for an increase compared with equivalent control and #p < 0.005 for a decrease compared with the non-treated control (MDA).

The small calcium-binding protein S100A4 was down-regulated to almost undetectable levels in all MDA/RLN2 stable transfectants when compared with the untransfected MDA-MB-231 cells or the MDA/EGFP vector controls (Figure 6a). T47D human breast cancer cells exposed to 100 ng/ml and 500 ng/ml rhRLN2 for 72 hours also responded with a reduction in S100A4 protein (Figure 6b) with 500 ng/ml rhRLN2 already being effective after 48 hours of exposure. Transient transfections with specific S100A4 siRNA probes were performed to knock-down S100A4 protein in MDA-MB-231 cells (Figure 7a). The aim was to investigate whether the observed reduction in cell motility caused by long-term exposure to rhRLN2 was mediated by the decrease in S100A4. S100A4 knock-down in MDA-MB-231 cells resulted in a reduction in cell motility (Figure 7b). In addition, 500 ng/ml rhRLN2 failed to increase cell motility after 24 hours of short-term exposure after S100A4 knock-down (Figure 7b). MDA-MB-231 cells transfected with a non-silencing siRNA probe or treated with lipofectamine only were able to respond to RLN2 with an increase in motility similar to untreated MDA-MB-231 cells (Figure 7b). These results suggested that relaxin's short-term effects on cell motility were dependent on S100A4 and that the down-regulation of S100A4 following constitutive exposure to RLN2 was responsible for the reduced cell motility and migration in stable MDA/RLNs transfectants.

### Long-term exposure to relaxin reduces xenograft tumour growth in vivo

**Figure 8:**
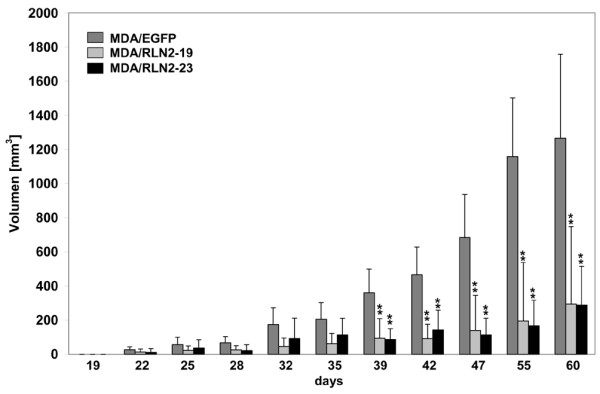
**MDA/RLN2 transfectants show reduced tumour growth *in vivo***. 2 × 10^7 ^cells/ml of the MDA/EGFP plasmid control transfectant and two of the MDA/RLN2 transfectants, MDA/RLN2-19 and MDA/RLN2-23, were injected subcutaneously in nude mice and the tumour growth rate (n = 10 tumours for each cell clone) was determined after 60 days of growth. All MDA/RLN2 xenografts showed slower tumour growth and smaller tumour sizes compared with the MDA/EGFP xenografts. Data are presented as mean ± SEM of tumour volume (mm^3^). A two-way ANOVA analysis was performed to determine statistical significance with p < 0.005 considered significant (**).

Of the five stable MDA/RLN2 cell clones, MDA/RLN2 clone 19 and 23 and MDA/EGFP were injected subcutaneously in nude mice and injection sites were monitored for a period of 60 days. MDA/EGFP tumours were palpable by week three and MDA/RLN2 tumours by week four. The mice had to be euthanized 60 days after injection of tumour cells because of rapid growth of the MDA/EGFP control tumours. By contrast, both MDA/RLN2 clones revealed much slower onset and progression of tumour growth as compared with MDA/EGFP control cells (Figure 8). All xenografts were removed at the end of the experiment and found to be encapsulated. Animals were inspected macroscopically and individual organs (brain, thyroid, submandibular glands, heart, lungs, diaphragm, liver, intestines, pancreas, spleen, prostate, bladder and kidney) were found to be free of metastases. To reveal bone metastases, mouse skeletons were subjected to X-ray analysis but also failed to show bone metastases in nude mice containing MDA/RLN2 or MDA/EGFP xenograft tumours.

### MDA/RLN2 xenograft tumours contain reduced S100A4 protein levels and show histological changes in differentiation

**Figure 9:**
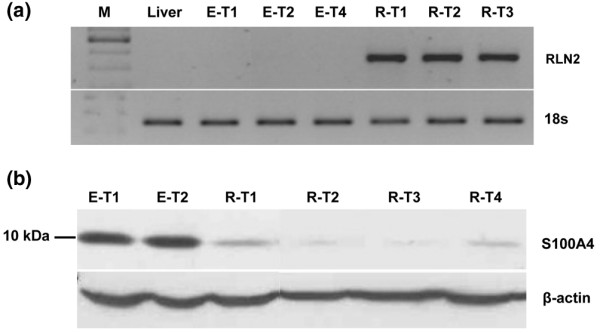
**S100A4 protein is reduced in MDA/RLN2 nude mouse xenografts**. (a) Total RNA was extracted from nude mice tumours and reverse transcriptase polymerase chain reaction (RT-PCR) analysis was performed for human RLN2. Representative results are shown for three tumours grown in mice injected with MDA/RLN2 transfectants (R-T1, R-T2, R-T3) and three tumours from mice injected with MDA/EGFP transfectants (E-T1, E-T2, E-T4). Mouse liver was used as negative control tissue. 18S transcript levels were used as reference. All MDA/RLN2 xenografts contained RLN2 transcripts. (b) A representative Western analysis is shown for four MDA/RLN2 tumours (R-T1, R-T2, R-T3, R-T4) demonstrating the down-regulation of S100A4 in all tumours when compared with tumours derived from MDA/EGFP transfectants (E-T1, E-T2). Beta-actin was used to control for protein loading of the gel.

**Figure 10:**
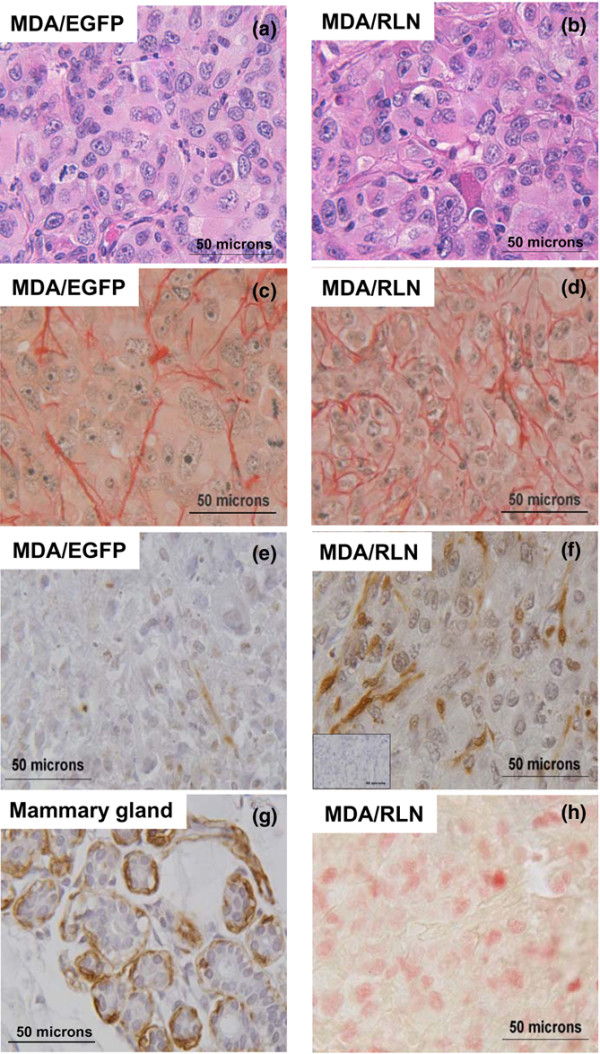
**MDA/RLN2 xenografts show immunohistological features of differentiation**. Tissue sections from MDA/EGFP (left column) and MDA/RLN2 tumours (right column) were stained with (a, b) haematoxylin and eosin and (c, d) picrosirious red showing smaller groups of tumour cells surrounded by collagen fibres in MDA/RLN2 tumours. Representative sections of the immunodetection of smooth-muscle actin are shown in (e) and (f). The primary antibody was substituted with an isotype control immunoglobulin and a representative control section is shown (insert in f). MDA/RLN2 tumours contained more smooth muscle actin positive cells then MDA/EGFP tumours. (g) A section of human mammary gland tissue was used as positive control and showed smooth muscle actin staining in the myoepithelial cells surrounding the alveoli. (h) Staining for the proliferation marker Ki67 revealed numerous positive nuclei in proliferative regions in the periphery of MDA/EGFP and MDA/RLN2 tumours, an example is shown here for MDA/RLN2. Magnification: ×400.

Relaxin transcripts were exclusively detected in the MDA/RLN2 xenografts (Figure 9a). Of the tumour tissues tested, expression of the 10 kDa S100A4 protein was strongly detectable in MDA/EGFP tumour tissues but was decreased to almost undetectable levels in all MDA/RLN2 xenograft tissues (Figure 9b) indicating the down-regulation of S100A4 during long-term exposure to RLN2 to be also effective *in vivo*. Histological analysis of the xenograft tissues confirmed the formation of encapsulated xenografts in all cases (data not shown) with MDA/EGFP and MDA/RLN2 xenografts containing areas with large tumour cells in the tumour periphery (Figure 10a, b) and necrotic areas in the centre of the tumours.

A TUNEL assay was performed to detect DNA fragmentation but all sections of MDA/EGFP and MDA/RLN2 tumours were devoid of apoptotic nuclei (data not shown). Picrosirius red staining of collagen fibres showed small groups of tumour cells surrounded by collagen in the MDA/RLN2 xenografts, whereas MDA/EGFP tumours appeared less structured with irregular and scarcely distributed collagen fibres (Figures 10c, d). Of all xenografts, MDA/RLN2 tumours displayed a higher number of cells positive for smooth muscle actin (Figures 10e, f). Human lobulo-alveolar breast tissue was used as a positive control and showed staining of myoepithelial cells surrounding the breast epithelial cells (Figure 10G). Xenotransplants of both MDA/RLN2 and MDA/EGFP clones revealed numerous cells positive for the proliferation marker Ki67 (Figure 10h) in the periphery of the tumours.

## Discussion

To the authors' knowledge, the current study is the first to describe distinct short-term and long-term effects of relaxin on the cell motility in oestrogen-independent human breast cancer cells. The human breast cancer cell line MDA-MB-231 does not express ERα and, thus, is a suitable model to study oestrogen receptor-independent effects of RLN2. MDA-MB-231 cells produced very low endogenous levels of RLN2. The relaxin receptor RXFP1 was expressed and functional as determined by the induction of intracellular cAMP on exposure to human recombinant RLN2. We employed MDA/RLN2 stable transfectants previously established in our laboratory and shown to produce the 18 kDa pro-RLN2. These stable MDA/RLN2 clones were shown here to secrete bioactive RLN2 and to retain the expression of RXFP1. Thus, MDA/RLN2 transfectants served as a cellular tool for the investigation of long-term relaxin exposure.

We show here that a 24 hour short-term exposure to rhRLN2 dose-dependently increased cell motility, as did conditioned media from MDA/RLN2 containing secreted RLN2. These data confirm previous findings by us and other authors who described relaxin as an enhancer of cancer cell motility/migration employing the human breast cancer cell lines MCF-7 and SKBr3 [19], human prostate cancer cells PC3 [20], human thyroid carcinoma cells [4] and human endometrial carcinoma cell lines HEC-1B and KLE [3]. Our results with siRNA knock-down of the relaxin receptor RXFP1 clearly demonstrated that the relaxin-induced increase in cell motility was RXFP1-dependent in MDA-MB-231 cells. The motility enhancing effect was paralleled by a small but significant reduction in cell proliferation demonstrating that relaxin did not act as mitogen in MDA-MB-231 human breast cancer cells. Relaxin was previously described to lack a growth stimulatory effect on the human endometrial cancer cell lines HEC-1B and KLE [3], human MDA-MB-435 and canine CF33.MT breast cancer cells [20]. Earlier *in vitro *studies found, that distinct relaxin concentrations and exposure times had a differentiation-promoting and growth-inhibitory effect of relaxin on MCF-7 cells after seven days of incubation [21,22].

We employed stable MDA/RLN2 transfectants to investigate the long-term effects of constitutive exposure to relaxin, which is most likely to be equivalent to the situation in breast tumours. Surprisingly, MDA/RLN2 transfectants showed a markedly reduced motility when compared with the EGFP-vector controls. Indeed, long-term exposure of MDA-MB-231 cells to rhRLN2 for seven consecutive days before motility assays also abolished the relaxin-induced increase in motility. This decreased motility in MDA/RLN2 was not the result of a down-regulation of RXFP1. Semi-quantitative RT-PCR analysis showed no reduction in RXFP1 transcripts in MDA/RLN2 transfectants and RXFP1 protein was detected by immunocytochemistry in MDA/RLN2 transfectants and MDA/EGFP vector controls. Interestingly, relaxin was previously shown to increase RXFP1 transcripts in a dose- and time-dependent manner in human decidual cells [45].

Earlier studies reported a relaxin-mediated enhanced cell migration and extracellular matrix invasiveness with several different cancer cell lines [3,4,17-20]. Our results demonstrated that long-term RLN2 exposure to relaxin negatively impacted on the invasive capacity of MDA-MB-231 cells as assessed by *in vitro *invasion assays using collagen A and laminin extracellular matrix components. We did not use the tumour matrix components of Matrigel to avoid the influence of numerous growth factors contained within this tumour matrix. The reduced invasive potential of MDA/RLN2 transfectants *in vitro *suggested that the reduction in cellular motility through down-regulation of S100A4 was the dominant mechanism of relaxin's long-term effect on MDA-MB-231 breast cancer cells. The small calcium-binding protein S100A4 was known to enhance cell motility by interacting with non-muscle myosin II and was described to selectively stimulate cell motility in mouse mammary carcinoma cell lines without increasing *in vitro *invasiveness [39,46,47]. We had previously reported that relaxin down-regulated S100A4 in MDA-MB-231 and in a stable MDA/RLN2 transfectant, thus, identifying S100A4 as a novel target gene for relaxin in breast cancer cells [26]. Here, we confirmed and extended this finding employing additional MDA/RLN2 transfectants, all of which having almost undetectable levels of S100A4 protein. We were able to demonstrate a similar down-regulation of S100A4 protein in ERα-positive T47D human breast cancer cells after 48 hours and 72 hours of RLN2 exposure. We have shown in this study that S100A4 siRNA knock-down abolished the ability of exogenous rhRLN to up-regulate cell motility in MDA-MB-231 cells. These findings demonstrated that the regulation of cell motility induced by relaxin was dependent on S100A4 and emphasise the importance of S100A4 as a relaxin target.

This down-regulation of the metastasis-promoting protein S100A4 is of high clinical importance. S100A4, also named metastasin, is a known promoter of tumour invasiveness and growth [38], and was demonstrated to increase invasiveness in mouse mammary carcinoma cells [37,48] and in a transgenic mouse model [34]. The expression of S100A4 in breast tumour tissues serves a prognostic marker for poor patient survival [29]. When injected subcutaneously in nude mice, all xenograft tumours derived from MDA/RLN2 transfectants contained reduced or almost undetectable levels of S100A4 protein when compared with the MDA/EGFP tumour tissues. This demonstrated for the first time that S100A4 is a relaxin target molecule *in vitro *and *in vivo *in human breast cancer cells. However, we were unable to detect a negative regulation of metastasis because all of the xenografts, including those of MDA/EGFP, failed to show macroscopic metastases. The 60 day time interval for this investigation was most likely to be too short for metastatic lesions to develop. The markedly reduced tumour size in MDA/RLN2 xenografts may have been a result of the reduced S100A4 production within the tumour cells.

Extracellular S100A4 was described to function as an angiogenic factor as observed in S100A4 transgenic mice [32] and *in vitro *when tested on human cerebro-microvascular endothelial cells [49]. Although MDA/EGFP and MDA/RLN2 xenografts showed large areas of necrotic tissue, on observation of haematoxylin and eosin-stained tumour sections there was some indication that there might be fewer blood vessels in MDA/RLN2 tumours. The reduced tumour size of MDA/RLN2 xenografts is potentially a combined result of the observed reduction in cell proliferation and the lack of an angiogenic stimulus by S100A4 secreted from the tumour cells. S100A4 was shown to bind to the tumour suppressor protein p53 and to co-operate with wild type p53 in apoptosis induction [50]. TUNEL assays performed on the xenograft sections failed to detect apoptotic nuclei in our MDA/RLN2 xenograft model with reduced tumour growth. Instead, relaxin may directly stimulate cell differentiation *in vivo*. We have detected an increased number of cells with positive staining for smooth muscle actin suggesting the presence of myoepithelial cells in MDA/RLN2 tumours. In addition, the collagen distribution in MDA/RLN2 tumours as determined by picrosirious red staining showed collagen to be more regularly distributed among strands of tumour cells in MDA/RLN2 tumours when compared with MDA/EGFP tumours. This may suggest a higher epithelial differentiation and organisation in the MDA/RLN2 tumours. Earlier studies showed that human MCF-7 breast cancer cell xenografts in oestrogen-treated nude mice revealed an increased differentiation response to systemic application of relaxin with the formation of differentiated epithelial- and myoepithelial-like cells [23]. Taken together, these data suggest that long-term exposure to relaxin may trigger differentiation-promoting and anti-invasive changes in breast cancer cells.

## Conclusion

In conclusion, the *in vitro *and *in vivo *down-regulation of S100A4 identifies a new and potentially clinically relevant property of relaxin in human breast cancer. Further studies are ongoing to identify the signalling pathways involved in the relaxin-induced regulation of S100A4.

## Abbreviations

BrdU = bromodeoxyuridine; cAMP = cyclic adenosine monophosphate; ERα = oestrogen receptor-alpha; FCS = fetal calf serum; IBMX = 3-isobutyl-1-methyl-xanthine; PBS = phosphate buffered saline; rhRLN = recombinant human RLN; RLN2 = H2 relaxin; RT-PCR = reverse transcriptase polymerase chain reaction; RXFP1 = relaxin/insulin-like family peptide receptor 1 (formerly: LGR7 = leucin-rich G protein-coupled receptor 7); siRNA = small interfering RNA; TUNEL = dUTP nick end labelling.

## Competing interests

The authors declare that they have no competing interests.

## Authors' contributions

Y.R. performed the majority of the experiments and analysed the data. C.H-V initiated and supervised the nude mouse *in vivo *studies and participated in the design and co-ordination. The principal investigator S.H-K was responsible for the overall concept of the study, for the design and interpretation of the data, the drafting and final approval of the manuscript.
